# TDP-43 Pathology and Prionic Behavior in Human Cellular Models of Alzheimer’s Disease Patients

**DOI:** 10.3390/biomedicines10020385

**Published:** 2022-02-05

**Authors:** Eva P. Cuevas, Alberto Rodríguez-Fernández, Valle Palomo, Ana Martínez, Ángeles Martín-Requero

**Affiliations:** 1 Department of Structural and Chemical Biology, Centro de Investigaciones Biológicas, Margarita Salas (CSIC), Ramiro de Maeztu 9, 28040 Madrid, Spain; e.p.cuevas@csic.es (E.P.C.); alberto.rodriguezf@edu.uah.es (A.R.-F.); vpalomo@cib.csic.es (V.P.); 2 Centro de Investigación Biomédica en Red de Enfermedades Neurodegenerativas (CIBERNED), Instituto de Salud Carlos III, 28031 Madrid, Spain; 3 Department of Molecular Biomedicine, Centro de Investigaciones Biológicas, Margarita Salas (CSIC), Ramiro de Maeztu 9, 28040 Madrid, Spain

**Keywords:** Alzheimer’s disease, extracellular vesicles, immortalized lymphocytes, vesicles, prionic behavior, TDP-43

## Abstract

Alzheimer’s disease (AD) is a neurodegenerative disorder for which there is currently no effective treatment. Despite advances in the molecular pathology of the characteristic histopathological markers of the disease (tau protein and β-amyloid), their translation to the clinic has not provided the expected results. Increasing evidences have demonstrated the presence of aggregates of TDP-43 (TAR DNA binding protein 43) in the postmortem brains of patients diagnosed with AD. The present research is focused on of the study of the pathological role of TDP-43 in AD. For this purpose, immortalized lymphocytes samples from patients diagnosed with different severity of sporadic AD were used and the TDP-43 pathology was analyzed against controls, looking for differences in their fragmentation, phosphorylation and cellular location using Western blot and immunocytochemical techniques. The results revealed an increase in TDP-43 fragmentation, as well as increased phosphorylation and aberrant localization of TDP-43 in the cytosolic compartment of lymphocytes of patients diagnosed with severe AD. Moreover, a fragment of approximately 25 KD was found in the extracellular medium of cells derived from severe AD individuals that seem to have prion-like characteristics. We conclude that TDP-43 plays a key role in AD pathogenesis and its cell to cell propagation.

## 1. Introduction

Alzheimer’s Disease (AD) is a neurodegenerative disorder marked by progressive impairment of cognitive ability, generally in later life, affecting more than 50 million people worldwide [1]. Age is the strongest risk factor for AD [2]. The prevalence of AD increased from 10% in individuals over 65 to 40% in subject over 80 years old [3], and it is expected to triple by 2050 due to an ageing population [4].

The disease begins with a decline in cognition followed by a number of other changes in brain functioning, including impairments in language and visual-spatial skills, and disorientation. This impairment in cognitive functions is due to anatomical atrophy of AD brains, which correlates with severe neuronal loss [5]. In particular, the lack of good coordination within structures like prefrontal cortex, hippocampus and amygdala seems to play a predominant role in cognitive deficits in the advanced age [6].

Based on age of onset, AD can be divided into early onset AD, and the more common, late-onset AD, in people over 65 years old, accounting for more than 90% of all AD cases [7]. Most cases of early onset AD have familial history of AD and are carriers of dominant autosomal mutations in three genes, *amyloid precursor protein (APP)*, *presenilin-1 (PSEN-1)*, *presenilin-2 (PSEN-2)* [8]. On the other hand, late onset AD is considered sporadic, although genetic risk factors have been identified [9].

AD is considered a multifactorial disease associated with a number of risk factors other than advanced age and genetic factors, such as environmental factors, diet, cardiovascular diseases, and diabetes, head injuries, and obesity among others [10,11,12]. Recently, the insulin resistance within the brain, the so-called type 3 diabetes (T3DM) has been shown to have a big impact in neurocognition in AD [13].

AD is characterized by two main neuropathological hallmarks: extracellular amyloid-β (Aβ) deposition in senile plaques and intracellular accumulations of hyperphosphorylated microtubule-associated tau, called neurofibrillary tangles (NFT) [14,15,16].

Based in the density of NFTs in various brain regions, AD cases have been classified in three different subtypes hippocampal-sparing, with lower NFT in hippocampus, limbic-predominant with lower cortical NFT, and the more frequent typical AD forms [17]. In addition to senile plaques and NFT, transactive response DNA-binding protein (TDP-43) has been found in limbic brain regions in up to 70% of late-onset AD patients [18,19]. Interestingly, TDP-43-positive inclusions in limbic brain regions have been recently considered as limbic-predominant age-associated TDP-43 encephalopathy (LATE) [20], suggesting that late-onset AD patients with TDP-43 proteinopathy may display concomitant LATE neurological changes.

TDP-43 was originally found to associate with frontal temporal lobar degeneration with ubiquitin inclusions (FTLD-U) and amyotrophic lateral sclerosis (ALS) [21]. These neurodegenerative diseases show a partial overlap in their clinical presentation, genetics and pathology [22] suggesting that they are part of a disease spectrum [23]. While FTD refers to a number of disorders with distinctive clinical phenotype caused by the loss of cortical neurons and basal ganglia, inducing changes in personality and language deficit [24]. ALS is characterized by the progressive loss of motoneurons, weakness of innervated muscles, and death by respiratory failure [25]. TDP-43 proteinopathy is the hallmark for the FTD/ALS spectrum. In addition, it is now recognized that TDP-43 pathology is present in other neurological diseases including AD and even in non-demented individuals [26,27].

The manner in which TDP-43 pathology induces neuron degeneration is not yet completely understood, and the role of TDP-43 in cognition remains elusive, since conflicting results had been reported [28,29]. Apparently TDP-43 pathology is most common in the medial temporal lobe, which may account for its robust association with episodic memory dysfunction [30].

TDP-43 is a predominantly nuclear protein, although it can shuttle between the nucleus and cytosol. It plays a variety of roles in RNA metabolism, including transcription, splicing, mRNA transport, mRNA stability through recruitment into stress granules (SGs), and microRNA biosynthesis [31,32]. Under pathological conditions, TDP-43 undergoes a number of posttranslational modifications (PTMs), including phosphorylation at serines 403/404 and 409/410, ubiquitination and abnormal cleavage to generate C-terminal fragments (CTFs). These posttranslational modifications lead to cytoplasmic accumulation and aggregation of TDP-43 [33]. A gain of toxic function in the cytoplasm as well as a loss of nuclear function seem to constitute TDP-43 disease mechanisms [34,35]. While TDP-43 pathological features of ALS and FTLD-TDP appear to be well established, the pattern of PTMs of TDP-43 in AD cases, as well as dissemination of TDP-43 proteinopathy remains to be fully elucidated.

The present work was undertaken to better understand the role and significance of TDP-43 in AD. To accomplish this goal, we assessed and compared pathological characteristics of TDP-43 of control and AD-derived immortalized lymphocytes. These lymphoblastoid cells lines had been extensively characterized in our laboratory, demonstrating that they display disease specific signature molecules [36,37,38], and therefore considered suitable experimental models for mechanistic and therapeutic studies. Moreover, we have recently demonstrated, specifically, the usefulness of lymphoblastoid cell lines to study TDP-43 homeostasis in FTLD-TDP or ALS [39,40,41].

The results presented here indicate an increase in TDP-43 fragmentation, as well as increased phosphorylation and aberrant localization of TDP-43 in the cytosolic compartment of lymphoblasts of patients diagnosed with severe AD. Moreover, a fragment of approximately 25 KDa was found in the extracellular medium of cells derived from AD individuals, that appears to be transported by extracellular vesicles (EVs) inducing TDP-43 pathological features and cytoskeletal changes in control cells. The data may suggest a role of these EVs containing fragmented TDP-43 in contributing to the propagation of AD disease in a prion-like manner.

## 2. Materials and Methods

### 2.1. Materials

RPMI 1640 culture medium (Cat#21875034 Gibco/Thermo Fisher, Waltham, MA, USA), DMEM culture medium (Cat#41965039 Gibco/Thermo Fisher, Waltham, MA, USA), Fetal Bovine Serum (FBS) (Cat#: F7524, Merck, Madrid, Spain), penicillin/ streptomycin (Cat#15140-122, Gibco/Thermo Fisher Waltham, MA, USA), polyvinylidene difluoride (PVDF) membranes for Western blots (Bio-Rad, Alcobendas, Madrid, Spain), enhanced chemiluminescence (ECL) system (Amersham, Uppsala, Sweden), Pierce BCA Protein Assay kit (Thermo Fisher, Waltham, MA, USA), protease inhibitor complete mini mixture (Roche, Mannheim, Germany).

Antibodies used in this study were from Santa Cruz Biotechnologies (Santa Cruz, CA, USA), Cell Signal (Danvers, MA, USA), Thermo Fisher (Waltham, MA, USA), Molecular Probes (Thermo Fisher), Bio-Rad (Alcobendas, Madrid, Spain) or Proteintech, (Manchester, UK) and are listed in Table 1.

### 2.2. Subjects

Healthy controls and patients were recruited from Hospital Doce de Octubre, Madrid Spain. The clinical diagnosis of probable AD was based on the criteria of the National Institute of Neurological and Communicative Disorders and Stroke and the Alzheimer’s Disease and Related Disorders Association (NINCDS-ADRDA) [42], and diagnosis required evidence of cognitive decline (neuropsychological test battery, clinical mental examination) as well as evidence of impairment in social or occupational function. The Mini-Mental State Examination was used to assess cognitive function [43]. Classification of mild, moderate and severe degrees of AD was performed using DSM-III-R criteria. Control individuals were usually age-matched family members of the patients, with no signs of neurological disease or cognitive decline. Demographic and clinical characteristics of subjects are provided in Table 2. This study was approved by the Ethic Committee of Clinical Investigation of the Hospital ‘12 de Octubre’(CEIC02506) and by the Spanish Council of Higher Research Institutional Review Board (15 March 2007) Informed consent from all subjects was obtained prior to their participation.

### 2.3. Isolation of Peripheral Blood Mononuclear Cells and Establishment of Lymphoblastoid Cell Lines

Blood samples (approximately 8 mL) were obtained through antecubital vein puncture in EDTA-treated tubes. Peripheral blood mononuclear cells (PBMCs) were isolated on Lymphoprep™ density-gradient centrifugation according to the instructions of the manufacturer (Axix-Shield Po CAS, Oslo, Norway).

Establishment of lymphoblastoid cell lines (LCLs) was performed in our laboratory, by infecting peripheral blood lymphocytes with the Epstein Barr virus (EBV) [44].

### 2.4. Cell Culture

Lymphoblastoid cells were grown in suspension in T flasks in an upright position, in approximately 10 mL RPMI 1640 medium that contained 10% (*v*/*v*) fetal bovine serum (FBS), and 1% penicillin/ streptomycin. U2OS cells were obtained from the American Type Culture Collection and grown in DMEM media, supplemented with 10% (*v*/*v*) fetal bovine serum and 1% penicillin/streptomycin. All cell lines were grown at 37 °C in a humidified 5% CO_2_ atmosphere.

### 2.5. Analysis of mRNA Levels by Quantitative Real-Time PCR

Total RNA extraction, cDNA synthesis and quantitative polymerase chain reaction (PCR) was done as detailed in the previous article [18]. Quantitative real-time PCR was performed with LightCycler^®^ 96 System (Roche, Mannheim, Germany) and the associated software using the manufacturer’s recommended conditions. Each reaction was performed in biological triplicates with 20 ng of cDNA by using FastStart Essential DNA Green Master (Roche, Mannheim, Germany). The sequences of the forward and reverse primers used are the following: for hTDP-43 FW: 5′-GAGAAGTTCTTATGGTGCAG-3′and RV: 5′-TGGCTTTGCTTAGAATTAGG-3′, for hRPS17 FW: 5′-CCATTATCCCCAGCAAAAAG-3′ and RV: 5′-GAGACCTCAGGA ACATAATTG-3′. Data analysis was based on the ΔΔCT method with normalization of the raw data to hRPS17 housekeeping gene.

### 2.6. Inmunological Assays

#### 2.6.1. Cell Extracts

To prepare whole cell extracts the lymphoblasts were seeded at initial density of 1 × 10^6^ cells × mL^−1^ and 24 h later, cells were harvested, washed in PBS and then lysed in ice-cold RIPA lysis buffer (50 mM Tris-HCl (pH 7.4), 150 mM NaCl, 5 mM EDTA, 15 mM MgCl_2_, 0.5% (vol/vol) sodium deoxycholate, 0.5% (vol/vol) NP-40 and 0.1% (vol/vol) SDS), containing 1 mM sodium orthovanadate, 1 mM phenylmethylsulfonylfluride (PMSF), 1 mM sodium pyrophosphate and protease inhibitor mixture. The protein content of the extracts was determined by de Pierce BCA Protein Assay kit.

#### 2.6.2. Western Blot Analysis

50-µg of protein from cell extracts or 35 µL of extracellular medium were fractionated on an SDS polyacrylamide gel, transferred to PVDF membrane and blocked with 5% non-fat milk. The antibodies used are listed in Table 1. Bands were detected with a chemiluminescent substrate detection system ECL using the Chemidoc Imaging System (Bio-Rad, Alcobendas, Madrid, Spain). Protein band densities were quantified using Image J software (National Institutes of Health, Bethesda, MD, USA).

#### 2.6.3. Confocal Laser Scanning Microscopy

Immunofluorescence analysis was performed on cells grown on coverslips. For the attachment of lymphoblastoid cells, the coverslips were previously coated with a solution of 0.025% Gelatin (Sigma, Madrid, Spain) for 30 min at room temperature followed by a solution of 1 mg/mL poly-L-lysine (Sigma, Madrid, Spain) diluted 1:50 in Borax buffer (Na_2_B_4_O_7_·10H_2_O 15 mM, pH 8.4) overnight at 37 °C.

Cells were fixed with 4% paraformaldehyde for 25 min, permeabilized with 0.1% Triton-X100 for 10 min and then blocked for 1 h at 37 °C with PBS-BSA 1%. Cells were incubated at 37 °C in a humidified chamber with the primary and secondary antibodies described in Table 1. Alexa Fluor-568 Phalloidin (1:1000, Molecular Probes, Waltham, MA, USA) was used for F-actin stain and nuclear staining was performed by incubation with DAPI (1:1000, Sigma). The preparations were mounted with Fluor Save reagent (Calbiochem, Madrid, Spain). Confocal microscopy analyses were performed using Leica TCS SP5 and a ×63 or ×100 oil immersion objective. Images were analysed using Leica Application Suite X (version 3.5.7.23225) and Image J software (version 1.53K).

### 2.7. Conditioned Medium Experiments

Conditioned medium from AD lymphoblastoid cells was collected after 72–96 h and centrifuged to eliminate cells and debris. Control lymphoblastoid cell lines or U2OS cells were cultured in the conditioned medium plus fresh medium in a 3:1 ratio for 72 h.

### 2.8. Extracellular Vesicles Isolation and Characterization

To generate conditioned medium for extracellular vesicles isolation, lymphoblasts were grown in RPMI medium with 10% of exosome-depleted FBS (Gibco, Waltham, MA, USA), instead of normal FBS. After 72 h, conditioned medium was recollected by centrifugation at 2000× *g* for 30 min to remove cells and debris. EVs were prepared with Total Exosome Isolation reagent (Invitrogen) following manufacturers’ recommendations. EV-containing pellet was resuspended in phosphate-buffered saline and stored at −20 °C until further analyses.

EV characterization was performed by nanoparticle tracking analysis (NTA) and Western Blot. Size distribution and quantification of EVs preparations were analyzed by measuring the rate of Brownian motion with a NanoSight LM10 system with a 630 nm laser (NanoSight, Wiltshire, UK), equipped with fast video capture and particle-tracking software. EVs samples were diluted 1:1000 in Hank’s balanced salt solution (HBSS), disaggregated and injected in the sample cubicle of the NanoSight. The mean of the number of particles acquired per milliliter was compared between AD and control cells and the measurements of EVs concentration (particles/mL) were normalized with the cell number from which conditioned medium containing EVs was recollected. For Western Blot analysis 20 µL of EVs were lysed with Laemmli buffer and denaturalized at 95 °C for 5 min. Western Blots were performed in parallel in EVs samples and cell lysates and the antibodies used are described in Table 1.

### 2.9. Statistical Analysis

Statistical analyses were performed using Graph Pad Prism software version 6 (La Jolla, CA, USA). Data are presented as means ± standard deviation (SD) of the mean. Statistical significance was estimated using one-way ANOVA followed by the Bonferroni test for multiple comparations, or, two-tailed Student’s *t*-test for statistical comparisons between groups. A “*p*-value < 0.05” was considered statistically significant.

## 3. Results

### 3.1. TDP-43 Pathology in AD Lymphoblasts

**Figure 1:**
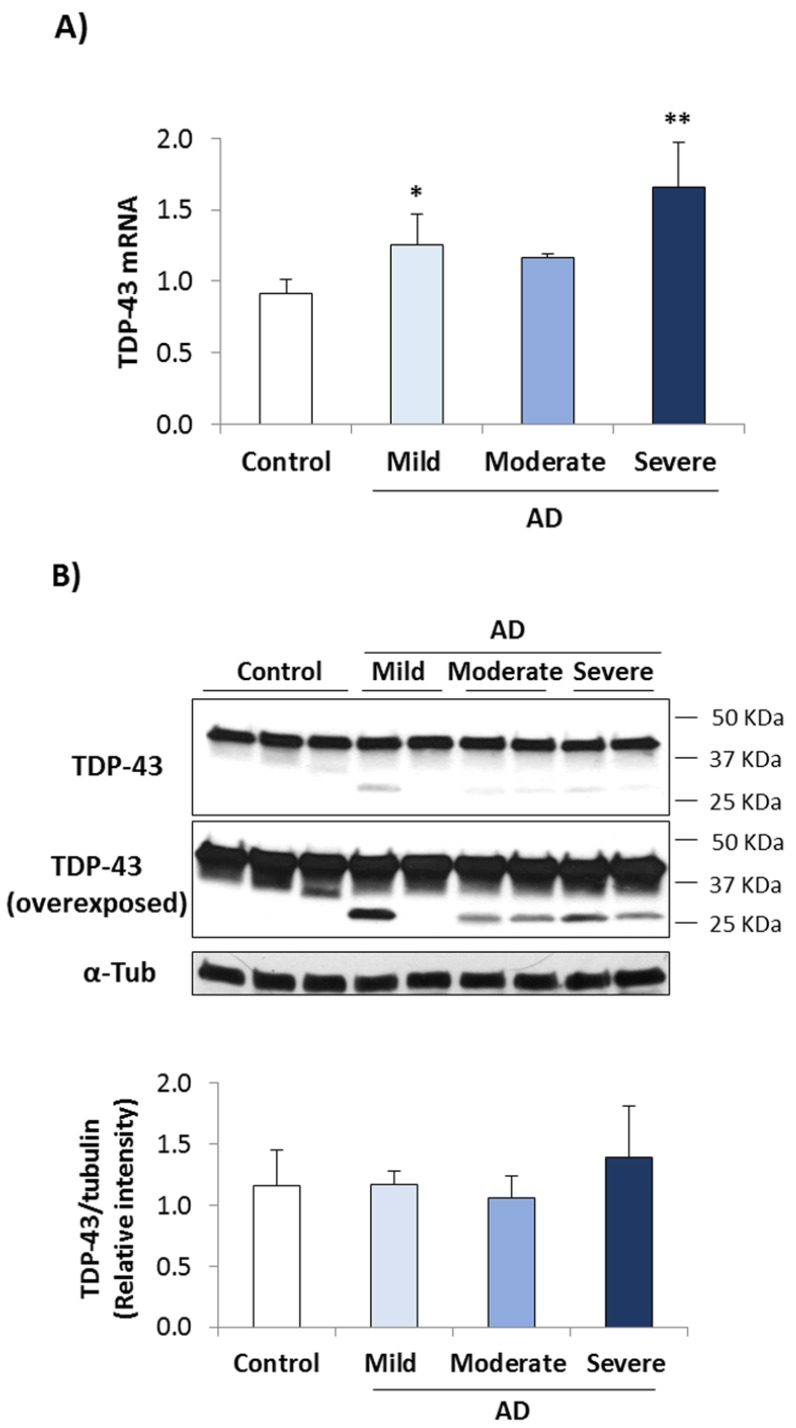
TDP-43 mRNA and protein expression levels in control and AD lymphoblasts. Immortalized lymphocytes from control and AD subjects (mild, moderate or severe patients), were seeded at an initial density of 1 × 10^6^ mL^−1^ and cultured in RPMI medium containing 10% FBS. 24 h later cells were harvested to isolate RNA and to prepare cell lysates. (**A**) TDP-43 mRNA expression levels were analyzed by quantitative RT-PCR. RPS17 levels serve as internal control. Results represent the mean ± standard deviation (SD) of two independent experiments performed on triplicate with four patients of each group. Statistical analysis was performed using 1-way analysis of variance (ANOVA; mRNA TDP-43: F_3.11_ = 8.733). * *p* < 0.05, ** *p* < 0.01 significantly different from control cells. (**B**) Western blot analyses (upper panels) and densitometric quantification (lower panel) were performed for the expression of TDP-43 protein. α-tubulin was used as loading control. A representative blot is shown and bars are the mean ± SD for each experimental group of two independent experiments.

To examine TDP-43 pathology in human lymphoblasts, we first analyzed the expression of TDP-43 mRNA and protein levels in immortalized lymphocytes from AD patients as a function of disease severity. Figure 1A shows an increase in TDP-43 mRNA levels in AD lymphoblasts and a trend to elevated protein levels in severe AD cases as compared with control individuals (Figure 1B).

Interestingly, a longer exposure of TDP-43 blots revealed the presence of a TDP-43 fragment of approximately 25–30 KDa perhaps due to TDP-43 cleavage, in most of the AD cases but not in control samples (Figure 1B).

**Figure 2:**
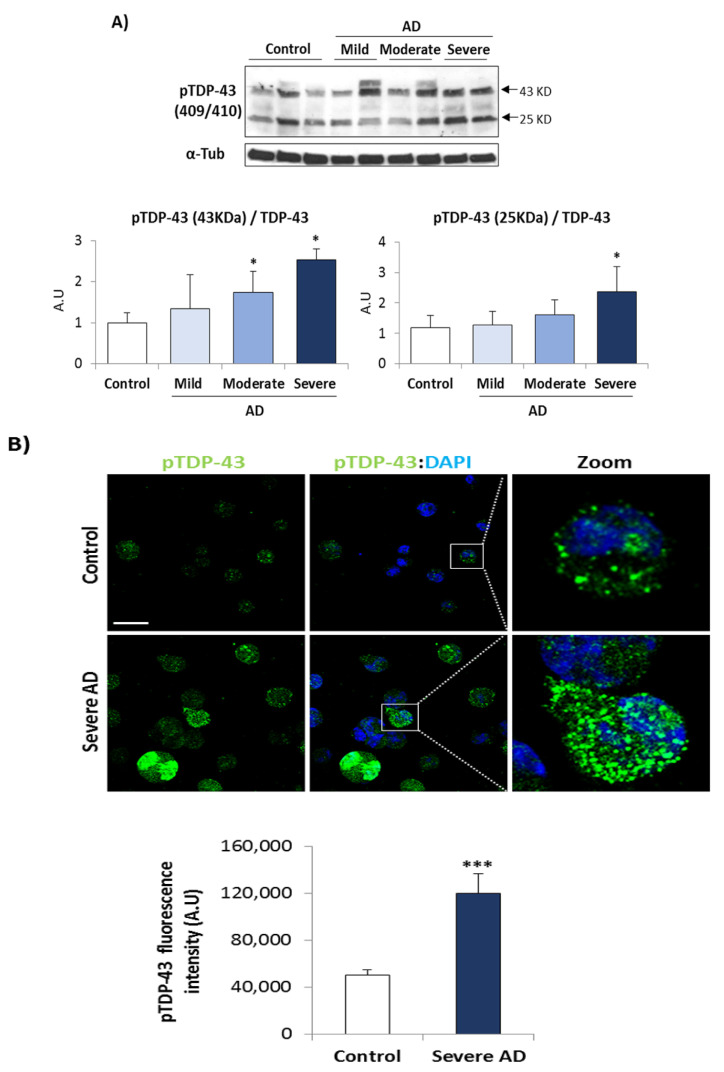
TDP-43 phosphorylation is increased in AD lymphoblasts. Immortalized lymphocytes from control and AD subjects (mild, moderate or severe patients) were incubated as described in legend of Figure 1, (**A**) Representative Western blot analysis of AD and control samples with pTDP-43 (Ser409/410) antibody. α-tubulin was used as loading control. Densitometric quantification of pTDP43 was normalized with total TDP-43 levels. Bars are the mean ± SD for each experimental group of two independent experiments. Statistical analysis was performed using 1-way analysis of variance (ANOVA; pTDP-43 (43KDa): F_3.11_ = 5.356, pTDP-43 (25KDa): F_3.10_ = 3.842). (* *p* < 0.05). (**B**) Immunofluorescence staining of pTDP-43 (green) in control or severe AD lymphoblasts. Nuclei were counterstained with DAPI (blue). Scale bar, 20 μm. Fluorescence intensity quantification was performed using Image J software in at least 4 fields of view (A.U, arbitrary units). Bars are the mean ± SD of two independent experiments. Statistical analysis was performed using the Student *t* test (*** *p* < 0.001).

We then, analyzed the phosphorylation status of TDP-43 in control an AD (mild, moderate, and severe) patients, using a phospho-specific antibody by Western blotting and confocal laser microscopy. As shown in Figure 2A a trend to an increase in phosphorylated levels of full length and truncated (25 KDa) TDP-43 was observed in AD lymphoblasts, regardless of AD severity, reaching statistical significance in moderate and severe AD cases. Figure 2B shows a significant increase in phosphorylated levels of TDP-43 protein, as assessed by immunofluorescence in lymphoblasts derived from severe AD patients.

**Figure 3:**
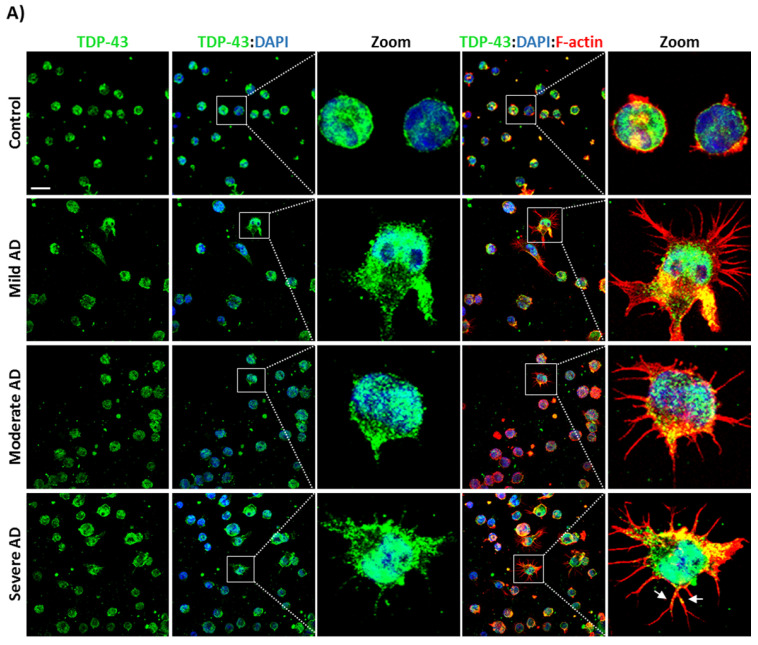

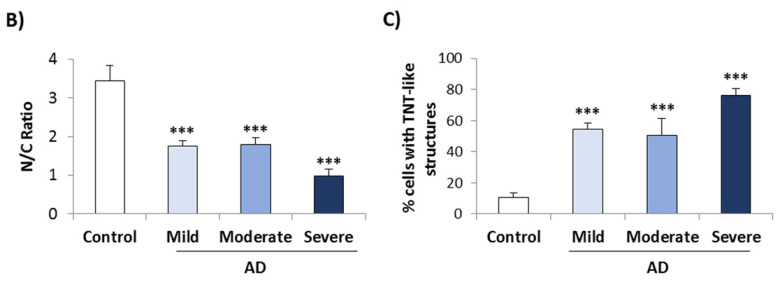
Lymphoblasts of AD patients display significative changes in TDP-43 subcellular localization and actin cytoskeleton organization. Lymphoblasts were seeded at 10^6^ cells × mL^−1^ and incubated in RPMI containing 10% FBS for 24 h. (**A**) Confocal immunofluorescence images of TDP-43 (green), F-actin (red) and DAPI staining (blue) in the indicated cells. Scale bar, 20 μm. (**B**) Fluorescence quantification and region of interest (ROI) selection was performed using Image J software. Nucleo-cytoplasm ratio was calculated using mean nuclear intensity divided by the mean cytoplasmic intensity. Measures were performed cell to cell in at least 4 fields of view. (**C**) The% of cells with F-actin protrusions (TNT-like structures) was measured in at least 4 fields of view. Bars are the mean ± SD of two independent experiments with at least two different patients of each type (control, mild AD, moderate AD, severe AD). Statistical analysis was performed using 1-way analysis of variance (ANOVA; N/C Ratio: F_3.10_ = 71.17% TNTs: F_3.10_ = 107.7). (*** *p* < 0.001).

Increased phosphorylation of TDP-43 had been associated with mislocalization of the protein in TDP-43 proteinopathies such as ALS and FTD [5]. For this reason, we seek to investigate whether nucleo-cytoplasmic TDP-43 shuttling is also perturbed in AD lymphoblasts. To this end we analysed the subcellular TDP-43 by immunofluorescence and laser confocal microscopy. As shown in Figure 3A, TDP-43 is mainly expressed in the nucleus of controls, as expected, whereas lymphoblasts from AD patients display a significant accumulation of TDP-43 in the cytosolic compartment and a significative reduction in the nucleo-cytoplasmic ratio (Figure 3B).

Moreover, immunofluorescence staining for filamentous actin (F-actin) with Alexa Fluor-568 phalloidin, revealed cytoskeletal abnormalities in AD lymphoblasts (Figure 3A) such as the increased formation of actin protrusions reminiscent of tunneling nanotubes (TNTs) or TNT-like structures. The percentage of AD cells showing these structures is significantly higher than in the case of control cells and seems to increase even more in severe AD cases (Figure 3C). We can observe also co-localization of F-actin and TDP-43 in the cytosolic compartment of AD cells and TDP-43 aggregates were found inside these tubular actin channels (Figure 3A, bottom zoom).

### 3.2. Conditional Medium from AD Lymphoblast Is Enriched in TDP-43 Fragments

**Figure 4:**
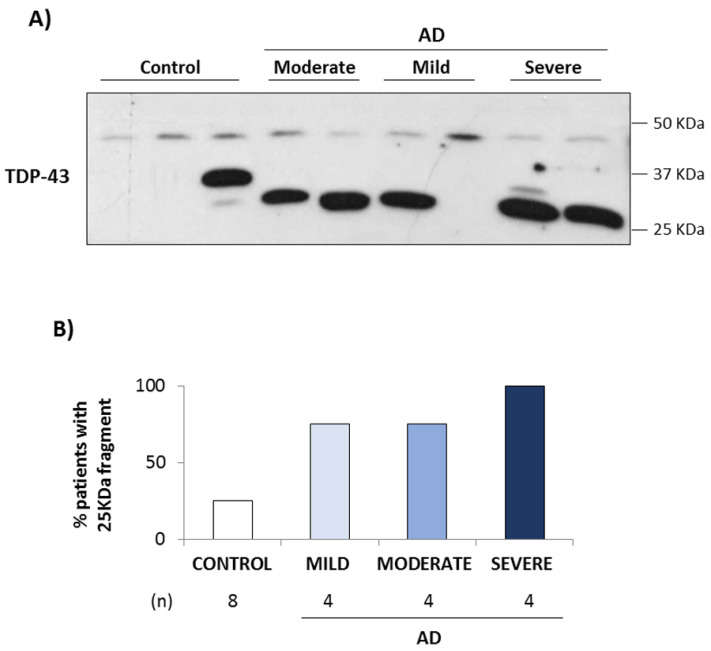
A 25 KDa fragment of TDP-43 is detected in the extracellular medium of AD lymphoblasts. (**A**) Representative Western blot of extracellular TDP-43 in the extracellular medium from control and AD lymphoblasts (mild, moderate and severe AD cases). (**B**) The% of samples with presence of 25 KDa-TDP-43 extracellular fragment was calculated in a total of 8 controls and 4 AD lymphoblasts of each type (moderate AD, mild AD, severe AD).

**Figure 5:**
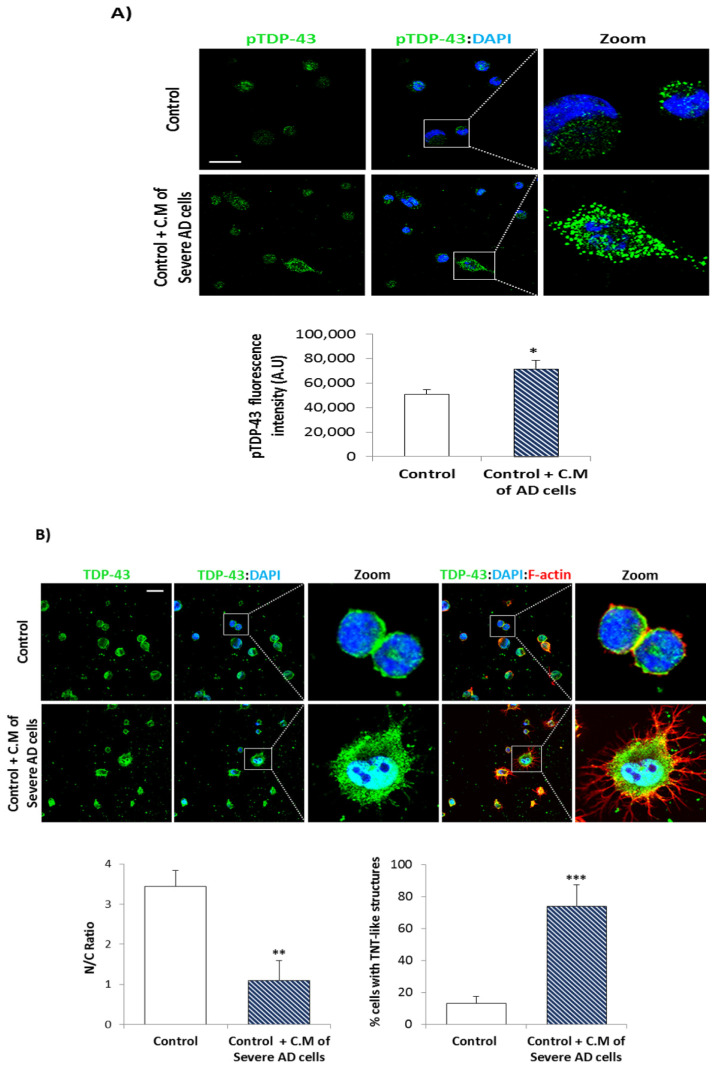
Conditioned medium (CM) of AD lymphoblasts induces TDP-43 pathology in control cells. (**A**) Immunofluorescence of pTDP-43 (green) and DAPI staining (blue) in control lymphoblasts cultured with CM of severe AD cells for 72 h versus untreated control cells. Fluorescence quantification was performed using Image J software in at least 4 fields of view. (**B**) Confocal immunofluorescence images of TDP-43 (green), F-actin (red) and DAPI staining (blue) in control lymphoblasts cultured in presence or absence of CM of severe AD cells for 72 h. Nucleo-cytoplasm ratio (mean nuclear intensity divided by the mean cytoplasmic intensity) and% of cells with F-actin protrusions (TNT-like structures) were measured in at least 4 fields of view using Image J software. (A.U, arbitrary units). Scale bars, 20 μm. Bars are the mean ± SD of three independent experiments Statistical analysis was performed using the Student *t* test. (* *p* < 0.05, ** *p* < 0.01, *** *p* < 0.001).

It has been documented that full-length and fragmented TDP-43 may be released in extracellular vesicles or exosomes in different neurological diseases. For this reason, we first preformed Western blot analysis looking for TDP-43 protein in the extracellular medium of control and AD lymphoblasts. Figure 4A shows a representative immunoblot carried out in the conditioned medium from control and AD lymphoblasts, where we can observe a TDP-43 fragment of approximately 25–30 KDa that appears in most AD cases, regarding of disease severity, and not in control lymphoblasts. Figure 4B shows the percentage of samples with this TDP-43 fragment in a total of 8 healthy individuals, and 12 AD patients (4 mild, 4 moderate and 4 severe). Of note a band of approximately 35 KDa was found in one control subject. We next performed conditioned medium (CM) experiments to investigate the possible transmission of TDP-43 pathology. CM from AD severe cases was collected after 72–96 h of culture and added to lymphoblasts derived from control individuals for 72 h. Immunofluorescence staining with anti-pTDP-43, revealed a significant increase in TDP-43 phosphorylation in control cells incubated with CM from AD cells (Figure 5A). Moreover, cytosolic TDP-43 accumulation, with a significative reduction in the nucleo-cytoplasmic ratio, was observed in control cells growing in CM of AD cells, when subcellular localization of TDP-43 was analyzed by immunostaining with anti-TDP-43 and DAPI (Figure 5B). We can also observe that in the control cells maintained in the CM of AD cells appear similar TNT-like structures (Figure 5B, right panels) to the ones previously found in AD lymphoblasts (Figure 3A, right panels).

**Figure 6:**
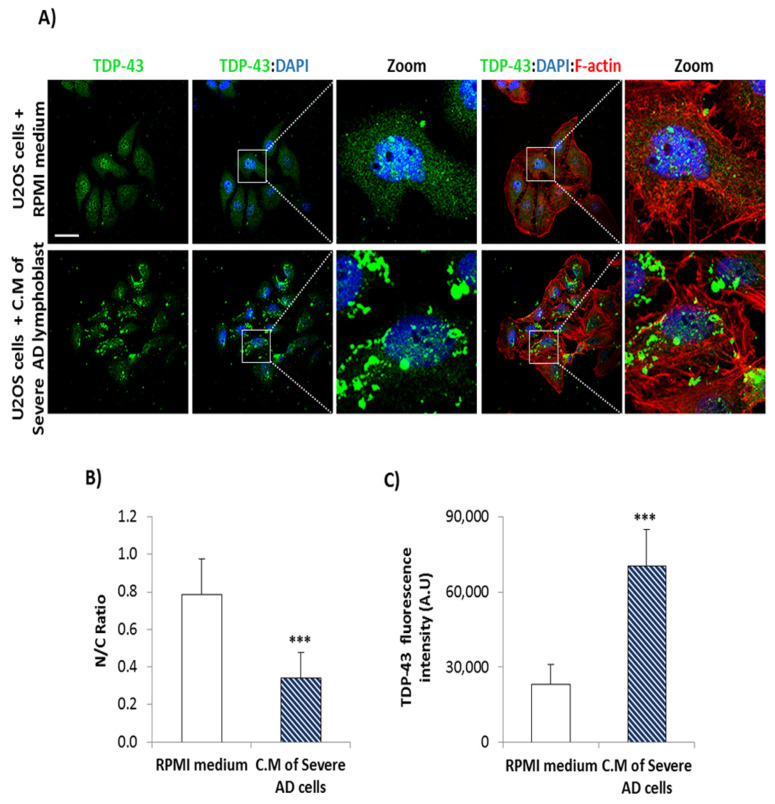
Conditioned medium (CM) of AD lymphoblasts induces TDP-43 pathology in U2OS osteosarcoma cells. (**A**) Confocal immunofluorescence images of TDP-43 (green), F-actin (red) and DAPI staining (blue) in U2OS cells treated with RPMI medium or CM of severe AD lymphoblasts for 72 h. Scale bar, 40 μm. (**B**) Nucleo-cytoplasm ratio (mean nuclear intensity divided by the mean cytoplasmic intensity) and (**C**) fluorescence intensity was measured cell to cell using Image J software in at least 4 fields of view. (A.U, arbitrary units). Bars are the mean ± SD of two independent experiments. Statistical analysis was performed using the Student *t* test. (*** *p* < 0.001).

Furthermore, CM from AD lymphoblasts induces also TDP-43 pathological changes, mislocalization and generation of TDP-43 aggregates in osteosarcoma U2OS cell line (Figure 6).

**Figure 7:**
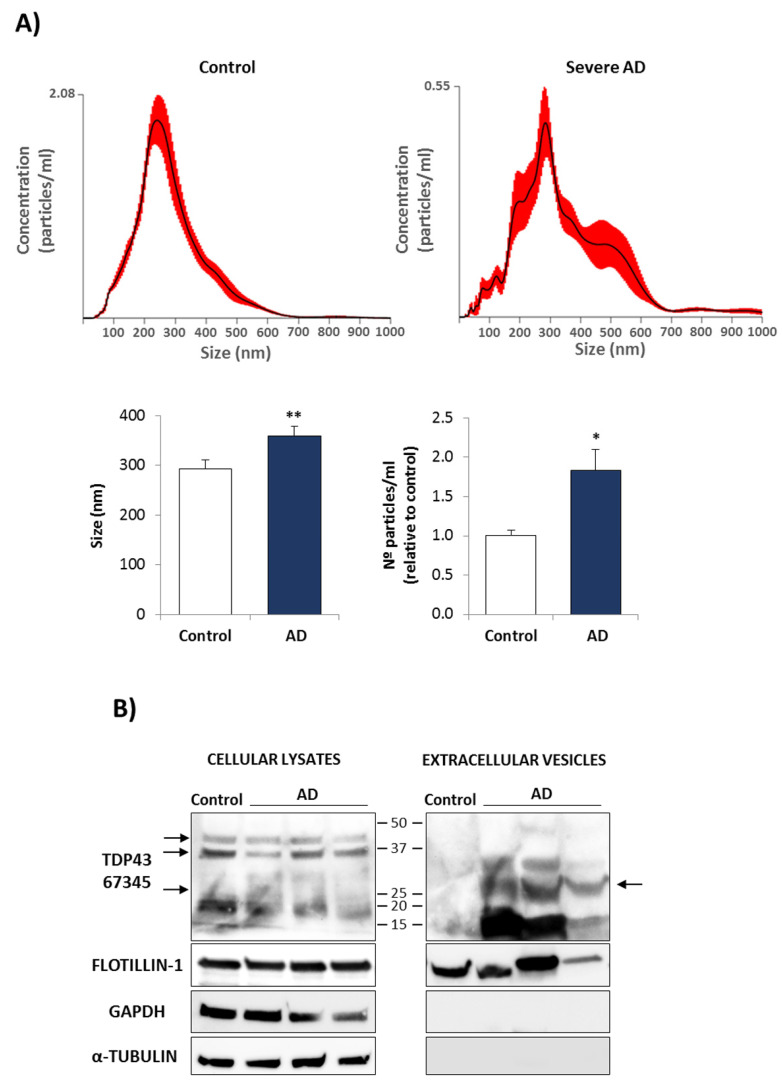
Extracellular Vesicles isolated from AD lymphoblasts showed an increase in size and concentration and were enriched in fragmented TDP-43. (**A**) Quantification of size and concentration of EVs released from control or severe AD lymphoblasts by NTA. Bars are the mean ± SD of three independent experiments. Statistical analysis was performed using the Student *t* test. (* *p* < 0.05, ** *p* < 0.01) (**B**) Western blot analysis of C-t TDP-43 in cellular lysates or extracellular vesicles of the indicated samples. α-tubulin and GAPDH were used as negative controls for EVs and Flotillin-1 as positive control. The arrow points to the 25 KD TDP43 fragment.

Finally, we isolated extracellular vesicles (EVs) from CM of control and AD cells, and NTA analysis was used to identify their size and concentration. As shown in Figure 7A, there was an apparent increase in particle size mean and concentration in severe AD cases. We then, performed Western blotting to detect the presence of pathological TDP-43 by using an antibody against the C-t epitope of TDP-43 (Proteintech, 67345-1-Ig), and anti-flotillin-1 in cell lysates and EVs from control and AD lymphoblasts (Figure 7B). GAPDH and tubulin antibodies were included as specificity control markers since they are not appreciably secreted in EVs. It is shown that the EV fractions isolated from severe AD lymphoblasts were particularly enriched in fragmented TDP-43, as compared to EVs from control cells.

## 4. Discussion

Over the past decade, TDP-43 deposition has been associated with an increasing number of neurodegenerative diseases [45] being the primary disease pathogenic factor such in the case of ALS, or a relevant protein that increase the pathogenicity present in others diseases. The latter is the case of AD, in where amyloid-β and tau pathologies may be exacerbated by the presence of TDP-43 [46,47]. However, the pathophysiological mechanisms through which TDP-43 mediates neurodegeneration appears complex, and deciphering these molecular processes seems critical for the development of effective therapies.

In this study we have examined some TDP-43 features in peripheral cells from AD patients as a function of disease severity. For this purpose, a comparative study on TDP-43 expression levels as well as TDP-43 phosphorylation pattern, protein fragmentation and subcellular localization was performed in lymphoblasts derived from control subjects and mild, moderate and severe AD patients.

The results herein presented show the presence of pathological features of TDP-43 in these easily accessible patient-derived cell lines, that could be considered systemic manifestations of the disease. Increased TDP-43 phosphorylation, protein cleavage, and cytosolic TDP-43 accumulation are more evident in lymphoblasts from severe AD patients. Moreover, higher expression of TDP-43 mRNA was observed in moderate and severe AD cases, which may be the result of increased self-regulation of TDP-43 expression, in order to maintain homeostasis in late stages of AD [48]. These observations are in line with previous reports that indicate that patients with abnormal TDP-43 immunohistochemistry show more severe hippocampal atrophy and score worse on the Clinical Dementia Rating Scale Sum of Boxes (CDR-SB), Mini-Mental State Examination (MMSE), and other neuropsychological tests [28].

TDP-43 pathological changes observed in AD lymphoblasts are similar to those found in lymphoblastoid cell lines derived from FTLD-TDP and ALS patients [39,40], despite the fact that distribution of TDP-43 pathology in AD seem to be distinct from other TDP-43 proteinopathies [26]. In AD, pathological TDP-43 is limited to the limbic system of the brain, being the amygdala the most vulnerable area [49]. This anatomical distribution of TDP-43 proteinopathy resembles what it is found in the disease recently named LATE (Limbic-predominant age related-TDP-43 encephalopathy) [20]. AD and LATE are often comorbid in individuals past age 80 years old.

Nowadays, there is an expanding field of research trying to elucidate how TDP-43 pathology may be mechanistically related with AD, especially with the limbic-predominant subtype, in which TDP-43 deposition is more frequent [50]. It is now recognized that TDP-43 deposition increases the risk for developing AD and influences the clinical features of dementia including cognitive deficits [26]. In addition, the fact that TDP-43 deposits are more abundant in the limbic system suggest a possible role of TDP-43 in the action control and emotion processing impaired in AD due to atrophy of prefrontal cortex and limbic system [6].

TDP-43 may participate in various pathogenic mechanisms underlying AD, such as amyloid β deposition, tau hyperphosphorylation, mitochondrial dysfunction, and neuroinflammation [51]. TDP-43 oligomers had been found to co-localize with tau and Aβ in AD [52] and it was reported that the presence of TDP-43 increases senile plaques load, perturbs amyloid clearance and induces synaptic loss [53,54]. On the other hand, TDP-43 seems to play a role in neurofibrillary tangle development [55]. Moreover, an inverse association between TDP-43 and tau was observed in post-mortem AD brains, which may the result of negative regulation of tau transcription by TDP-43 [56].

TDP-43 pathological features, such as increased phosphorylation, cleavage, aggregation and cytoplasmic accumulation are believed to impaired neuronal function [33]. It is still a matter of debate whether the clearance of TDP-43 from the nucleus causes a loss of normal TDP-43 functions, that leads to neurodegeneration, or, alternatively, the retention of TDP-43 in cytoplasmic aggregates could induce neurodegeneration through a toxic gain of function [57]. Recently, some protein kinase inhibitors such as CK1, CDC7 and TTBK1 inhibitors have shown to recover TDP-43 homeostasis in immortalized lymphoblast from ALS and FTLD-TDP [41,58,59]. These new drug candidates emerge as potential new therapies for correct the TDP-43 pathology present in AD samples, offering also the possibility to be included in multi-target approaches.

Since TDP-43 protein contains a prion-like domain in its C-terminus, it was hypothesized that TDP-43 toxic species could be transmitted intercellularly [60]. Therefore, we look for the presence of TDP-43 in the extracellular medium. Our results showed that the extracellular medium derived from AD was particularly enriched in a TDP-43 fragment of approximately 25 KDa, although full-length TDP-43 is also present. Conditioned medium (CM) from AD cells induced, in control lymphoblasts, an increase in TDP-43 phosphorylation as well as TDP-43 cytoplasmic redistribution. Moreover, CM induced changes in cytoskeleton, promoting the appearance of actin protusions in control cells, similar to those found in severe AD lymphoblasts. These protusions resemble the so-called tunneling nanotubes-like structures (TNTs), reported to play an important role in intercellular spread of prions [61]. Our finding that TDP-43 aggregates were detected inside these structures in AD lymphoblasts suggests that they can participate in dissemination of pathological TDP-43 proteinopathy.

Finally, it is worth highlighting that fragmented TDP-43 (25 KDa) and, in less amount, full-length TDP-43 were detected in EVs isolated from AD lymphoblasts. EVs have been shown to participate in the communication and transfer of cargo among different cell types within the CNS [61], modulating the physiological state of the recipient cells. Thus, a similar mechanism seems to be operative in regulating intercellular communication in non-neuronal cells from AD patients.

## 5. Conclusions

We confirmed that TDP-43 pathology is present in peripheral cells from AD patients, being exacerbated with disease severity. Thus, protein kinase inhibitors able to modulate TDP-43 emerge as promising agents for AD including those patients in more advanced stages. Furthermore, the addition of these treatments to those able to decrease amyloid-β may open new hopes for AD therapy. A prion-like disease propagation of TDP-43 pathology via actin protrusions and secretion of fragmented TDP-43 in EVs is in line with the finding that conditioned medium from AD cells provoked TDP-43 phosphorylation and cytoplasmic accumulation in control cells. Although further work is warranted for a better understanding of mechanisms involved in disease dissemination and to elucidate the possible interactions of pathological TDP-43 with other AD-associated molecules, amyloid-β and tau, for patient stratification and development of novel therapeutic strategies, this last discovery support TDP-43 potential therapies as disease-modifying agents able to modulate the neurodegeneration.

## 6. Limitations and Future Studies

The results of this study are somehow limited by the scarce number of patients included in the analysis. The patient-sample set should be increased to validate these results. We believe that the analysis of the complex interactions among TDP-43, amyloid-β, and tau may open new areas of therapeutic interest for the treatment of this devastating disease, mainly based in multitarget approaches. Research aiming to study the influence of protein kinase inhibitors able to recover TDP-43 homeostasis, in pathogenic mechanisms underlying AD pathology and spreading is ongoing in our laboratory.

## Figures and Tables

**Table 1:** Primary and secondary antibodies used in the analysis.

**Primary Antibody**	**Species**	**Dilution** **(WB/IF)**	**Supplier (Catalog#)**
TDP-43	Rabbit	1:1000/na	Proteintech (10782-2-AP)
TDP-43	Mouse	1:1000/1:1000	Proteintech (67345-1-Ig)
p(Ser409/410)- TDP-43	Rabbit	1:500/1:1000	Proteintech (22309-1-AP)
α-tubulin	Mouse	1:5000/na	Santa Cruz (23948)
GADPH	Rabbit	1:1000/na	Cell Signal (5174)
Flotillin-1	Rabbit	1:1000/na	Thermo Fisher (PA5-97756)
**Secondary Antibody**	**Immunological Procedure**	**Dilution**	**Supplier (Catalog#)**
Goat anti-mouseIgG HRP conjugate	WB	1:7000	Bio-Rad (1706516)
Goat anti-rabbitIgG HRP conjugate	WB	1:7000	Bio-Rad (1706515)
Anti-mouse Alexa 488	IF	1:1000	Molecular Probes (A-11001)

na: not apply; WB: Western blot; IF: immunofluorescence.

**Table 2:** Summary data of the study population.

Cell Lines	n	Age		Gender	
		Mean	Range Age	Male	Female
CONTROL	8	68 ± 12	52–83	5	3
AD					
Total	12	74 ± 7	60–84	6	6
Mild	4	74 ± 6	66–80	2	2
Moderate	4	79 ± 5	73–84	2	2
Severe	4	70 ± 7	60–75	2	2

Values expressed as mean ± standard deviation. Healthy control individuals, no sign of neurological disease; AD. Patients with a diagnosis of probable Alzheimer’s disease; mild AD (DSM-III-R, Mini Mental State Examination (MMSE) score between 18–24, moderate AD (MMSE: 10–18), and severe AD (MMSE: <10).

## Data Availability

The datasets analyzed during the present study are available from the corresponding authors on reasonable request.
